# Novel Helical Trp- and Arg-Rich Antimicrobial Peptides Locate Near Membrane Surfaces and Rigidify Lipid Model Membranes

**DOI:** 10.1002/anbr.202300013

**Published:** 2023-04-15

**Authors:** Saheli Mitra, Mark Coopershlyak, Yunshu Li, Bhairavi Chandersekhar, Rachel Koenig, Mei-Tung Chen, Brandt Evans, Frank Heinrich, Berthony Deslouches, Stephanie Tristram-Nagle

**Affiliations:** Biological Physics Group Physics Department Carnegie Mellon University Pittsburgh, PA 15213, USA; Biological Physics Group Physics Department Carnegie Mellon University Pittsburgh, PA 15213, USA; Biological Physics Group Physics Department Carnegie Mellon University Pittsburgh, PA 15213, USA; Biological Physics Group Physics Department Carnegie Mellon University Pittsburgh, PA 15213, USA; Biological Physics Group Physics Department Carnegie Mellon University Pittsburgh, PA 15213, USA; Biological Physics Group Physics Department Carnegie Mellon University Pittsburgh, PA 15213, USA; Biological Physics Group Physics Department Carnegie Mellon University Pittsburgh, PA 15213, USA; Biological Physics Group Physics Department Carnegie Mellon University Pittsburgh, PA 15213, USA; Center for Neutron Research National Institute of Standards and Technology Gaithersburg, MD 20878, USA; Department of Environmental and Occupational Health University of Pittsburgh Pittsburgh, PA 15261, USA; Biological Physics Group Physics Department Carnegie Mellon University Pittsburgh, PA 15213, USA

**Keywords:** antimicrobial peptides (AMPs), circular dichroism (CD), elasticity, Gram-negative bacteria, Gram-positive bacteria, minimum inhibitory concentration (MIC), X-ray diffuse scattering (XDS)

## Abstract

Antibiotics are losing effectiveness as bacteria become resistant to conventional drugs. To find new alternatives, antimicrobial peptides (AMPs) are rationally designed with different lengths, charges, hydrophobicities (H), and hydrophobic moments (μH), containing only three types of amino acids: arginine, tryptophan, and valine. Six AMPs with low minimum inhibitory concentrations (MICs) and <25% toxicity to mammalian cells are selected for biophysical studies. Their secondary structures are determined using circular dichroism (CD), which finds that the % α-helicity of AMPs depends on composition of the lipid model membranes (LMMs): gram-negative (G(−)) inner membrane (IM) >gram-positive (G(+)) > Euk33 (eukaryotic with 33 mol% cholesterol). The two most effective peptides, E2–35 (16 amino acid [AA] residues) and E2–05 (22 AAs), are predominantly helical in G(−) IM and G(+) LMMs. AMP/membrane interactions such as membrane elasticity, chain order parameter, and location of the peptides in the membrane are investigated by low-angle and wide-angle X-ray diffuse scattering (XDS). It is found that headgroup location correlates with efficacy and toxicity. The membrane bending modulus $K_{\text{C}}$ displays nonmonotonic changes due to increasing concentrations of E2–35 and E2–05 in G(−) and G(+) LMMs, suggesting a bacterial killing mechanism where domain formation causes ion and water leakage.

## 1. Introduction

Overuse and poor stewardship of antibiotics have accelerated antimicrobial resistance (AMR) into a global health calamity, exacerbated by a limited antibiotic discovery pipeline.^[1–4]^ It was estimated that, globally, more than 4 million deaths worldwide were associated with multidrug-resistant (MDR) bacterial infections in 2019.^[5]^ Therefore, it is crucial to search for new alternatives for antibiotics that are effective without being toxic or invoking AMR. In this context, antimicrobial peptides (AMPs) from natural sources, which are effector molecules of innate immunity, and synthetic variants have become a potent alternative.^[6,7]^ As AMPs are amphipathic (part hydrophilic and part hydrophobic), they can easily bind to lipid bilayers, which have a hydrophilic headgroup and hydrophobic interior. Traditional antibiotics mainly attack bacterial cell walls by disrupting the peptidoglycan layer,^[8,9]^ DNA replication,^[10]^ protein synthesis,^[11]^ or folic acid metabolism.^[12]^ By contrast, AMPs kill bacteria by perturbing the membrane in a nonspecific manner. The membrane perturbation takes many forms including pore formation,^[13–15]^ which can be as a “barrel-stave”^[13,16]^ or “toroidal”,^[17,18]^ interfacial activity,^[19]^ bilayer thinning,^[20–22]^ segregation of the charged lipids in the membrane,^[23]^ or solvation which is also known as the “carpet” model.^[24]^ Further, Chen et al. have proposed the “membrane discrimination model”, in which membrane lipid composition selects the mode of AMP action, i.e., the same helical AMP may form a barrel-stave in a eukaryotic membrane and a carpet in a bacterial membrane.^[25]^ Unlike mammalian cell membranes, bacterial membranes are rich in anionic lipids; thus, cationic AMPs selectively interact with them.^[26,27]^ Due to this lipid membrane focus instead of a metabolic pathway, AMPs induce bacterial resistance and toxicity more slowly than do traditional antibiotics.^[28]^ Thus, AMPs could provide a much-needed alternative to conventional antibiotics.^[18,29,30]^

**Figure 1.**
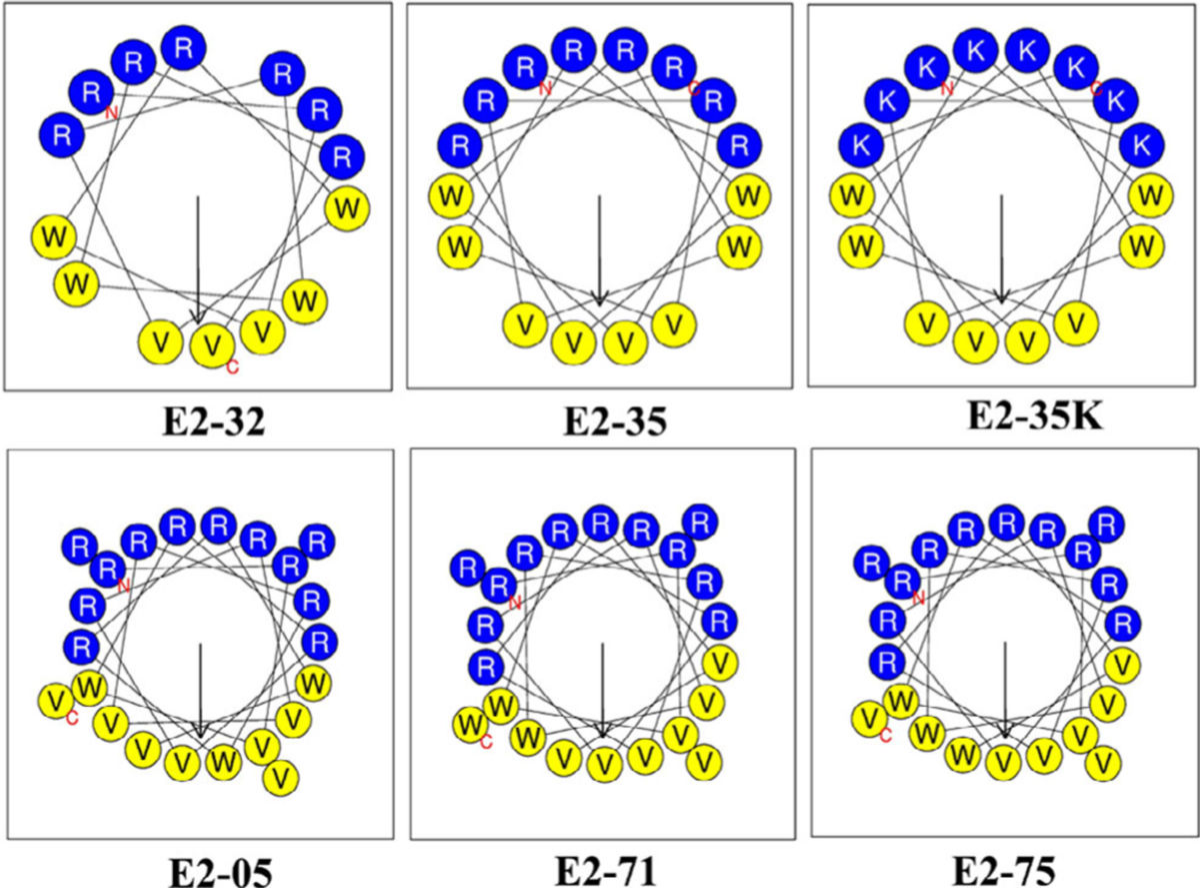
Helical wheel projections depict the distribution of amino acids in engineered peptides. The diagrams are prepared using the HELIQUEST web server (https://heliquest.ipmc.cnrs.fr/). The arrows represent the direction of the hydrophobic moment μH, which is a measure of helical amphipathicity.

Recent developments have shown that cationic AMPs can be engineered to increase their selectivity by choosing specific combinations of amino acids, such as positively charged arginine (Arg, R) residues on the helix polar face and hydrophobic valine (Val, V) residues on the helix nonpolar face.^[31]^ Other modifications are to increase peptide chain length^[32,33]^ and introduce tryptophan (Trp, W) on the hydrophobic side of the peptide.^[34]^ In nature, tritrpticin and indolicidin are two AMPs enriched with W. It has been shown by Deslouches et al. that the inclusion of the W residue not only enhances the antimicrobial potency against a broad spectrum of G(−) and G(+) bacteria, but also decreases their sensitivity to salt and serum.^[32]^ For example, the W-rich, 24 residue de novo engineered peptide WLBU2 has a broader spectrum antimicrobial activity compared to other available AMPs like LL-37, polymyxin B, and colistin.^[35]^ Another challenge in AMP design is to increase antibacterial potency without increasing the risk of host toxicity, i.e., increase the therapeutic index (maximum tolerated dose/minimum effective therapeutic dose). In this context, Deslouches and co-workers follow a rational framework to design the peptides in the present study, where one or two Rs (or lysines (Ks)) are followed by a hydrophobic dimer (either two Vs or V and W) (see Table 2 for peptide sequence). Assuming a perfect helix, this design ensures that all the positively charged residues are on one face, while the hydrophobic residues are on the opposite face. It is known that the exclusive use of W in the hydrophobic domain enhances not only antimicrobial functions but also host toxicity due to its high hydrophobicity and bulky indole ring.^[32,36]^ Thus, to mitigate toxicity to eukaryotic cells, V was added to the hydrophobic domain due to its substantially lower hydrophobicity compared to W. W residues are placed either at the C-terminus, or in the middle of the hydrophobic region as shown in Figure 1.

Numerous biophysical approaches have been carried out on AMPs.^[37–40]^ From our lab, an X-ray diffuse scattering (XDS) study confirmed a change in membrane elasticity and lipid chain ordering of lipid membranes in the presence of polymyxin E (colistin) that suggested a mechanism of action involving lipid domains.^[41]^ An additional study found that both WLBU2 and D8-WLBU2 (a stereoisomer) produced changes in elasticity similar to colistin.^[42]^ Further, a major distinguishing feature of AMPs is their ability to adopt secondary structures (α-helix, β-sheet, β-turn, and random coil) when in contact with bacterial membranes that could be crucial for their in vivo efficacy.^[43]^ We have systematically optimized the secondary structure of WLBU2 to form an amphipathic α-helical structure in order to enhance its antibacterial potency.^[44]^

Recently, Xiang et al. have engineered multiple W-based libraries of cationic peptide antibiotics, consisting of a total of 86 peptides containing only R or K, W, and V, and compared them to several antibiotic controls.^[45]^ Their study showed that these engineered AMPs displayed broad efficacy against MDR bacteria.^[45]^ In the present work, we chose five of these AMPs, plus a derivative where R→K, which are displayed as helical wheels in Figure 1. We obtain the secondary structure of these six AMPs in G(−) (Gram-negative), G(+) (Gram-positive) bacterial, and Euk33 (eukaryotic with 33 mol% cholesterol) cell lipid model membranes (LMMs), and the peptide/lipid interactions of a subset (E2–05, E2–35, and E2–35K). The peptides were rationally designed, which varies the length, hydrophobicity (H), hydrophobic moment (μH), helicity, overall net charge, and position of W.^[45,46]^

**Figure 2.**
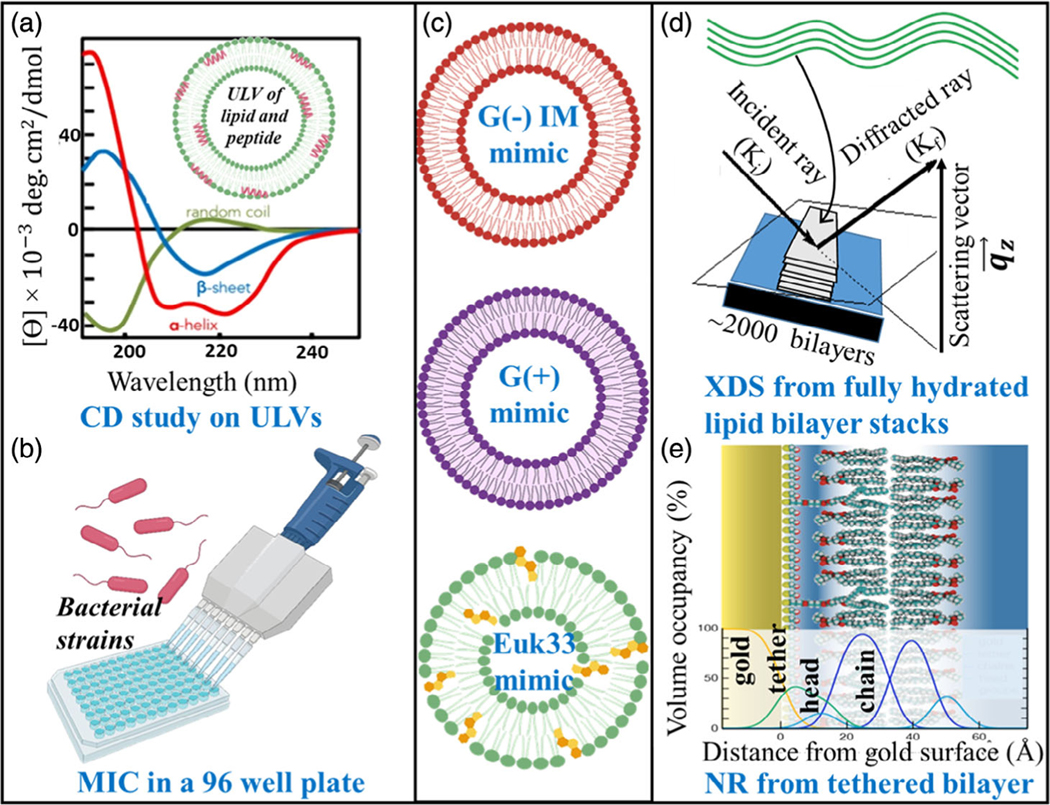
Schematic representation of different experimental techniques used in this article: a) CD study on large unilamellar vesicles (ULVs) of G(−) IM (POPE/POPG/TOCL (7:2:1 molar ratio)), G(+) (POPG/DOTAP/POPE/TOCL (6:1.5:1.5:1)), and Euk33 (POPC/ESM/POPE/cholesterol (15:4:1:10) (33 mol% cholesterol)) LMMs containing AMPs. b) Antibacterial assay in a 96-well plate. c) Schematic of ULVs used in the biophysical studies. d) XDS from fully hydrated lipid bilayer stacks on silicon wafers to obtain material properties, lipid chain order, and structure of the lipid LMMs in the presence of AMPs. e) NR from a single tethered bilayer to study the position of the AMPs in the LMMs. The figure was created with BioRender.

Circular dichroism (CD) measurements were used to study AMP secondary structures and explore correlations with activity. Antibacterial activity and cytotoxicity were determined by in vitro microbiological assays. XDS was used to probe membrane structure, the location of AMPs in the three LMMs, as well as membrane rigidity and lipid chain order. Neutron reflectometry (NR) confirmed AMP location in the G(−) inner membrane (IM) LMM. A schematic representation of the different experimental techniques used in this work is shown in Figure 2.

## 2. Results and Discussion

### 2.1. Toxicity to Bacteria

**Figure 3.**
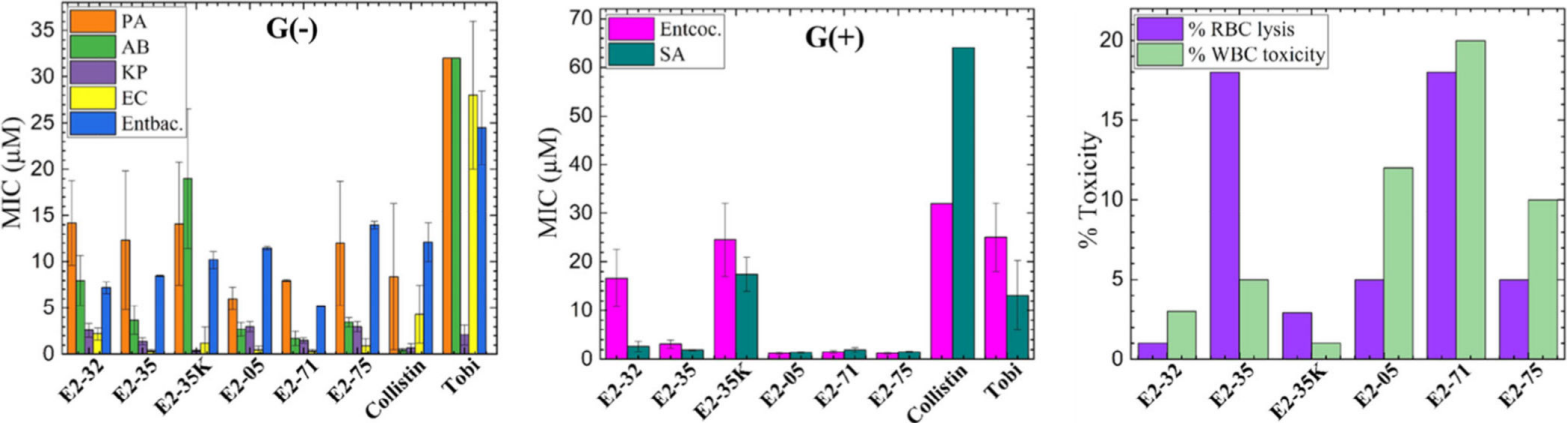
Antibacterial activity and toxicity of selected E2 peptides and controls. Selected peptides were examined for MIC against G(−) and G(+) MDR isolates from UPMC. Abbreviations: G(−): *Pseudomonas aeruginosa* (PA), *Acinetobacter baumannii* (AB), *Klebsiella pneumoniae* (KP), *Eschericihia coli* (EC), *Enterobacter* (Entbac). G(+) *Enterococci faecalis* (Entcoc.) and *Staphylococcus aureus* (SA). The MICs are the average of four strains of each type of bacteria. % Red blood cell (RBC) lysis at 32 μM and % toxicity at 16 μM against freshly isolated human white blood cells (WBCs) were determined by live–dead stain incorporation using flow cytometry. Maximum test concentrations (MTCs) are limited to 16 or 32 μM to ensure each peptide is available for iterative structure–function testing against large panels of antibiotic-resistant clinical isolates. Data are representative of 2–3 experimental trials. Error bars are corresponding to standard error of the mean values, $\sigma =\text{Std.}\;\text{dev.}/\sqrt{N}.\;\text{Std.}\;\text{dev.}$ are calculated by combining the standard deviations for each bacterial species, $\sigma_{\text{Ave}}=\surd \left({\left(\sigma_{A}\right)}^{2}+{\left(\sigma_{\text{B}}\right)}^{2}+{\left(\sigma_{\text{C}}\right)}^{2}\ldots \right)/N$.

Minimum inhibitory concentration (MIC) values for the six peptides are plotted in Figure 3. The MICs represent the average of four different strains of each type of bacteria; MICs were redetermined after publication of ref. [45]. The G(−) bacteria and strains are *Pseudomonas aerginosa (PA231, PA232, PA239, PA243), Acinetobacter baumannii (AB273, AB274, AB275, AB276), Klebsiella pneumoniae (KP542, KP548, KP550, KP552), Escherichia coli (EC541, EC543, EC546, EC549)*, and *Enterobacter (EA62, EC544, EC545, EA547)*. The G(+) bacteria and strains are *Enterococci (EF500, EF671, EF672, EF673)* and *Staphylococcus aureus (SA461, SA462, SA463, SA561)*. Remarkably, most peptides were broadly active against both G(−) and G(+) bacterial species.

It is evident in Figure 3 that there is a large variability in MIC values across bacterial species. G(−) bacteria contain negatively charged lipopolysaccharide (LPS) on their surface and peptidoglycan in the periplasmic space. Each type of G(−) bacteria contains different O-antigens with different numbers of carbohydrates or negative charges in the LPS lipids, or different lengths of lipoteichoic acid in G(+) bacteria which will affect the surface charge.^[47]^ We suggest that species specificity is distinguished by varying surface charge.^[48,49]^

### 2.2. Toxicity to Eukaryotic Cells

All peptides were examined for lytic activity against RBCs and WBCs as an indication of toxicity to eukaryotic cells. The percent RBC lysis was tested at the maximum concentration (32 μM), while % WBC toxicity was tested at 16 μM. The data in Figure 3 suggest that the most efficient peptides in killing bacteria are also the most toxic to eukaryotic cells. Notwithstanding, the % RBC lysis and WBC toxicity are <25% in all cases, which is an important criterion for AMP selection.^[45]^

In an attempt to understand which physical properties are most important for both bacterial killing efficiency and toxicity to eukaryotic membranes, we plotted μH or H versus MIC or versus % toxicity (Figure S1, Supporting Information), and μH/H versus MIC or versus % toxicity (Figure S2, Supporting Information). See the Supporting Information for these results and discussion.

### 2.3. Secondary Structure

AMPs are typically relatively short (i.e., fewer than 100 amino acids) and exhibit amphipathic properties because they usually contain cationic and hydrophobic residues. Despite these common characteristics, they are highly diverse with respect to their primary, secondary, and tertiary structures.^[50,51]^ The α-helical secondary structure, with hydrophilic residues aligned along one side and hydrophobic residues along the opposite, allows for an optimal interaction of peptides with membranes.^[52]^ It is well known that certain amino acids favor adopting a helical structure while others destabilize it. Mangoni et al. improved peptide activity against G(+) and fungus by replacing glycine with proline in Temporin L (TL).^[53]^ Deleting glycine or substituting with leucine in the N-terminus of melittin correlates well with its increased helicity and antimicrobial activity.^[54]^ Magainin 2-derived peptides which are designed to have higher α-helicity also promote antibacterial activity.^[55]^ Although these findings demonstrate the importance of the helical structure of AMPs, exceptions exist. For example, the D8 form of WLBU2, containing eight valines as the D-enantiomer, displayed a random coil structure in G(−) LMMs, unlike WLBU2’s mainly helical structure. Yet, both AMPs had similar efficacy at killing bacteria.^[20,56]^

**Figure 4.**
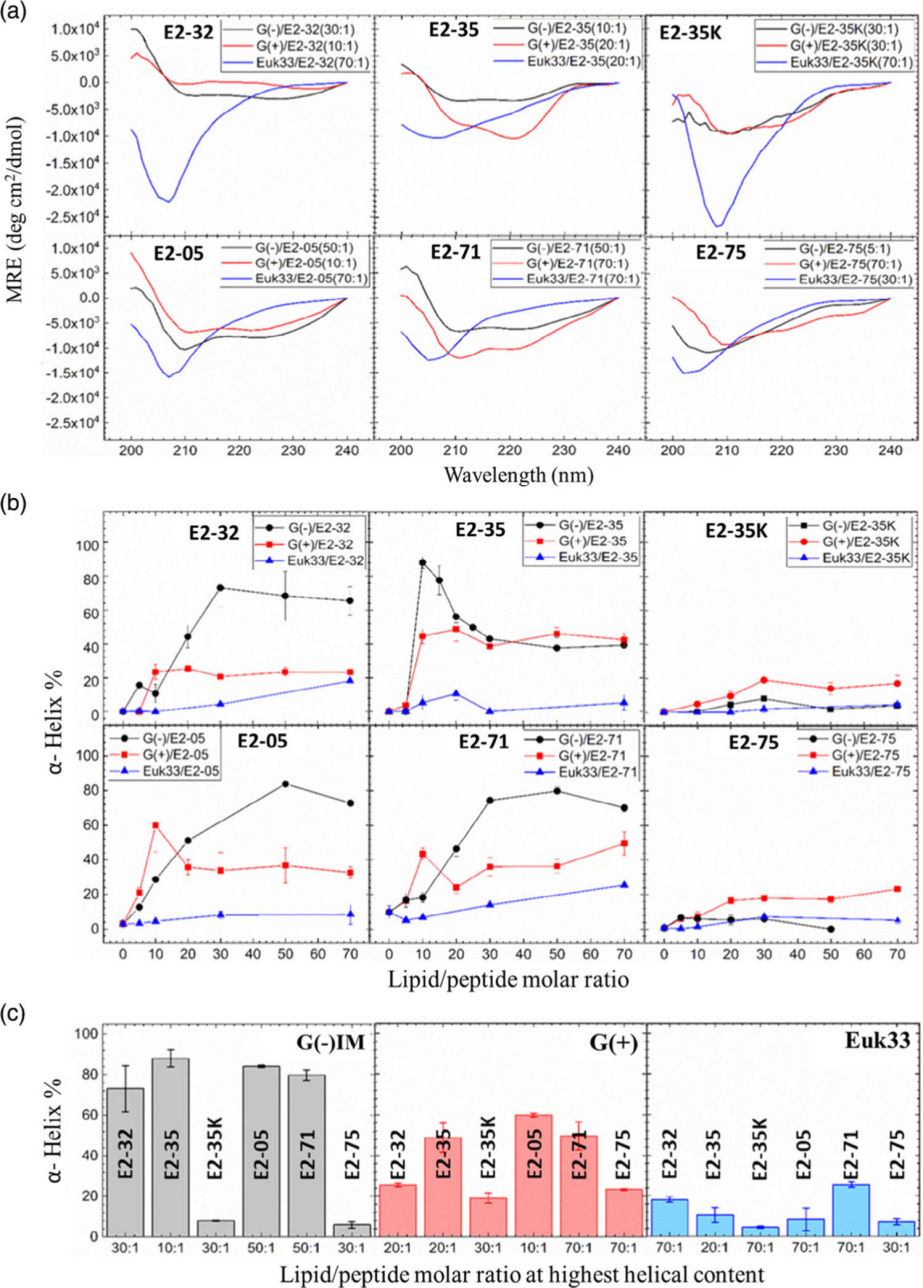
a) MRE results of six peptides in three different LMM ULVs: G(−) IM (black), G(+) (red), and Euk33 (blue). Lipid/peptide molar ratios shown in legend induce the maximum helical content. b) % α-helix versus lipid/peptide molar ratio. Colors as in (a). c) Summary of AMPs’ helical content in three LMMs: G(−) IM (gray), G(+) (red), and Euk33 (blue). The lipid/peptide molar ratio (in parentheses) is for the highest helical content. Standard deviations represent 3–4 fitting results using shape analysis.

To measure helicity in the present study, CD solution studies were used to obtain mean residue ellipticities (MRE) for all six peptides in three different LMM ULVs (Figure 4a). Four secondary structural motifs (α-helix, β-sheet, δ-turn, and random coil) were fit to the ellipticity data using Levenberg–Marquardt least squares fitting as described in Experimental Section. MRE data for lipid/peptide molar ratios with the highest α-helical content are shown in Figure 4a. A plot of the % α-helix versus lipid/peptide molar ratio is shown in Figure 4b. A comparison of the maximum % α-helicity for all six peptides is shown in Figure 4c. A summary of the percentage of all four secondary structural motifs of the peptides is shown in Table S1–S18, Supporting Information. These results indicate that peptide α-helicity depends on the composition of the LMMs: G(−)>G(+)>Euk33. E2–35 and E2–05 were predominantly helical in G(−) IM (85%–90%) and in G(+) (50%−60%) LMMs, while the substitution of R with K (E2−35→E2−35K) dramatically decreased helicity. E2–71 and E2–75 each contain 22 amino acid residues and the same amino acid composition with 3 Rs located close to the interface in the helical wheel design (Figure 1). However, E2–75 displayed a dramatic decrease in helicity compared to E2–71 due to a single valine quite close to the 3 Ws, which had the effect of unfolding the helix. The lipid/peptide molar ratio at which the maximum helical content occurred varied for all peptides (Figure 4b). All peptides had a lower helical content in Euk33 membranes, suggesting that cholesterol inhibits helicity. Other investigators have also found that the % helicity of AMPs is reduced in the presence of cholesterol in the membrane.^[57–60]^

**Figure 5.**
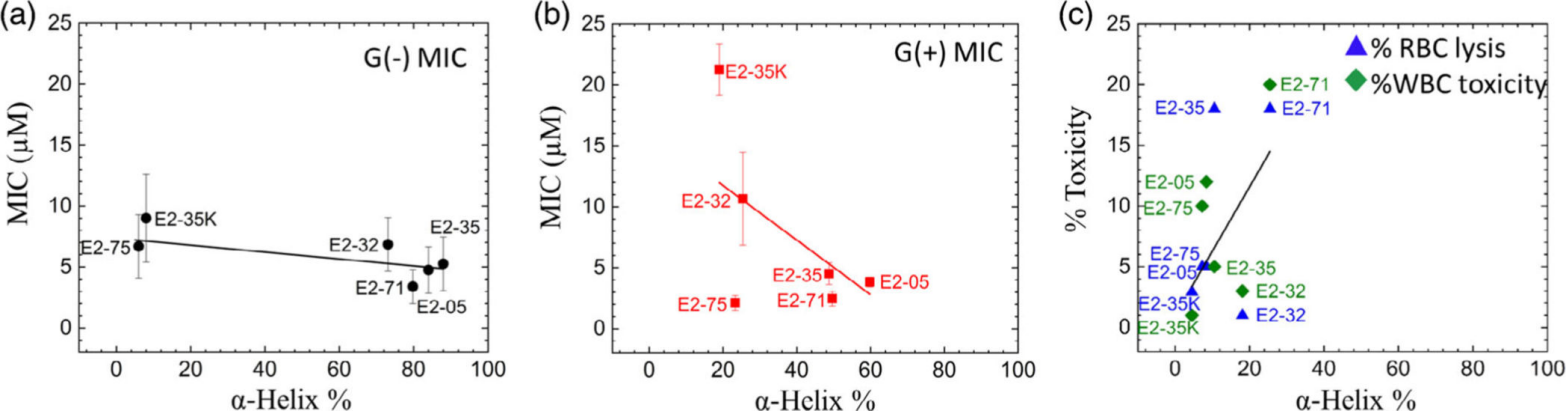
a) MIC versus % α-helix for AMPs in G(−) LMMs (black circles). b) MIC versus % α-helix for AMPs in G(+) LMMs (red squares). The standard deviations were corresponding to average MIC values. c) % toxicity caused by AMPs in RBCs (blue triangles) and WBCs (green diamonds). % α-helix was determined in Euk33 LMMs. Straight lines are linear fits to the data.

Figure 5a plots MIC versus % α-helix for six AMPs in G(−) IM LMMs. As shown, there was a very slight negative slope, indicating that higher helical content correlates with lower MIC (more efficient). Figure 5b shows MIC versus % α-helix for six AMPs in G(+) bacteria, where the negative slope is steeper. Panel c shows % toxicity of both RBCs and WBCs versus the % α-helix for six AMPs. The higher α-helical content of E2–35, E2–05, and E2–71 correlates with a lower MIC (Figure 5a,b) and higher toxicity (Figure 5c), suggesting that consideration of helicity is essential for designing new AMPs. However, as mentioned above, % helicity may not be a reliable measure to assess the efficacy of AMPs in general because other efficient AMPs rely on different secondary structures, such as β-sheet.^[61]^

### 2.4. Membrane Elasticity and Lipid Chain Order Parameter

**Figure 6.**
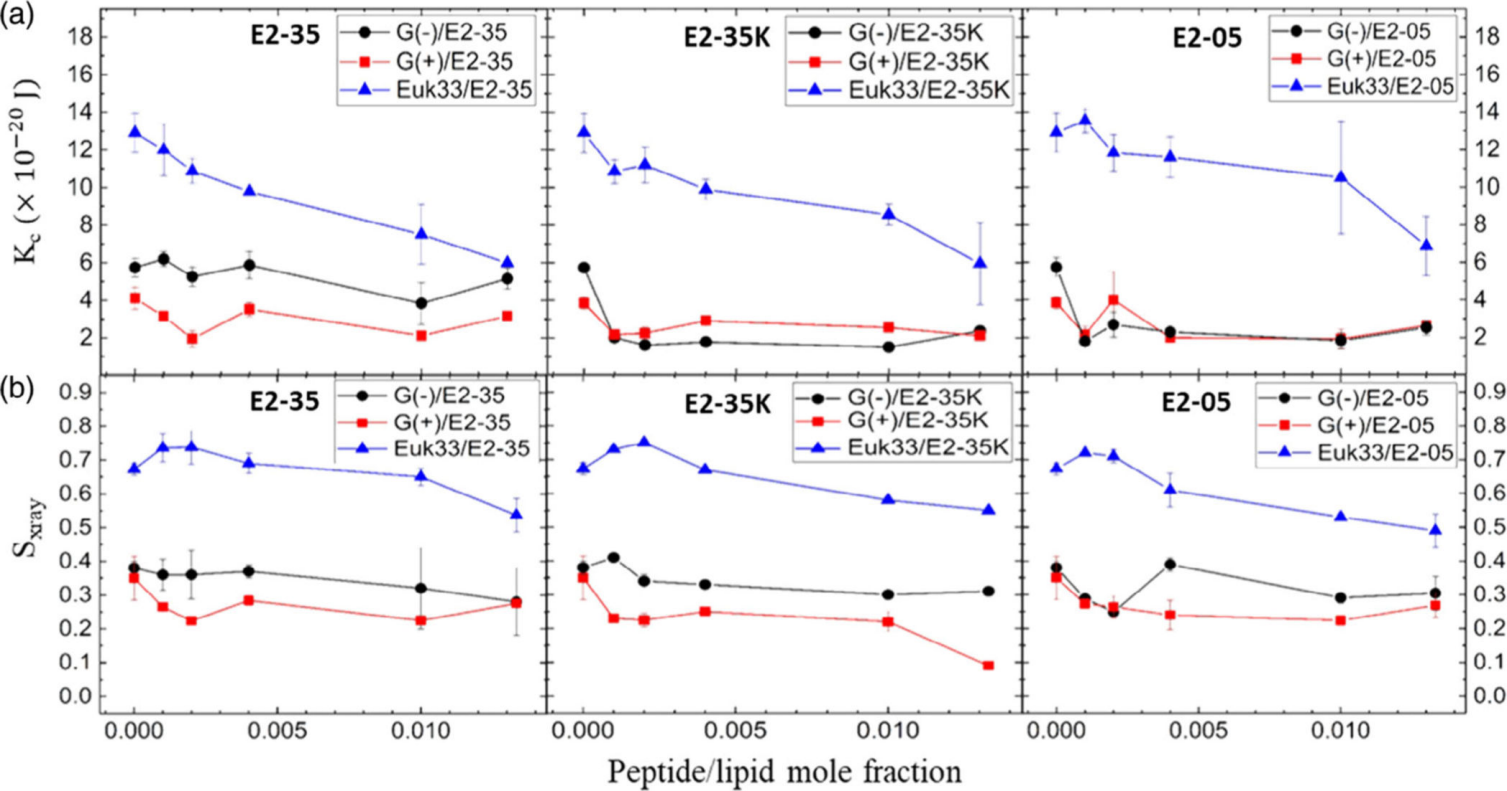
a) Bending modulus ($K_{\text{C}}$) of G(−) IM (black circles), G(+) (red squares), and Euk33 (blue triangles) LMMs interacting with three AMPs as shown. b) Chain order parameter ($S_{\text{X-ray}}$) of three AMPs with LMMs (colors as in (a). The standard deviations are from duplicate or triplicate samples.

While six peptides were studied for microbiological activity, only three were utilized for XDS, due to the considerable time required to collect and analyze synchrotron XDS data. We chose E2–35 and E2–05 because they are both effective at killing bacteria but they have different lengths (see Table 2), and we chose E2–35K because its chemical structure is identical to that of E2–35, except all 8 Rs are replaced by Ks. Figure 6a shows the elastic bending modulus parameter ($K_{\text{C}}$) of G(−) IM (black),G(+) (red), and Euk33 LMMs (blue) in the presence of all three AMPs. A higher value of $K_{\text{C}}$ indicates a stiffer membrane and a lower value indicates a softer membrane. $K_{\text{C}}$ is highest for Euk33 LMM because it contains 33 mol% of cholesterol. Previous studies from our lab indicate that cholesterol interacts preferentially with the saturated palmitoyl chain in POPC and POPE, making these membranes stiffer and also ordering the lipid acyl chains.^[62–64]^ The higher value of $K_{\text{C}}$ for G(−) control compared to G(+) control is due to the higher PE content as shown by Dupuy et al.^[41]^ The changes in $K_{\text{C}}$ follow variable nonmonotonic behavior with increasing concentration of AMP only for E2–35 and E2–05. Similar nonmonotonic behavior was observed with WLBU2 and D8 in G(−) IM and G(+) LMMs by Kumagai et al.^[42]^ and with colistin in G(−) IM LMM by Dupuy et al.^[41]^ We previously suggested that membrane stiffening could result from the interaction of the AMPs with the PE component of the membranes, whereas membrane softening could result from interaction with the negatively charged lipids, PG and cardiolipin, which were tested separately.^[41]^

This could lead to domain formation with different bending moduli in the bacterial LMMs. At the interface of these domains, defects could arise allowing leakage of ions and water through the domain wall, which would dissipate the bacterial membrane potential. The idea of domain formation of different material moduli has been reported by Polyansky et al.^[65]^ and Lopez-Cascales et al. using molecular simulation studies.^[66]^ Clustering of the anionic lipids (PG and cardiolipin) in bacterial membranes has been proposed to play a pivotal role in cell division, membrane protein function, and the action of antimicrobial agents.^[23,67–69]^ E2–35K, which is ineffective in bacterial killing, did not display the nonmonotonic $K_{\text{C}}$ behavior.

In Figure 6b, acyl chain order ($S_{\text{X-ray}}$) is plotted versus peptide/lipid mole fraction. Higher values of $S_{\text{X-ray}}$ signify ordered lipid acyl chains while lower values signify disordered lipid acyl chains. The Euk33 control, containing 33 mol% of cholesterol, had the most ordered chains. All the AMPs in Euk33 LMMs caused initial ordering at low concentrations and then disordering at higher concentrations, indicating that lipid chain order was not significantly different for E2–05 and E2–35 compared to E2–35K, and may be unrelated to their different toxicities. G(+) control LMM had the most disordered chains compared to the other control LMMs, similar to its lowest $K_{\text{C}}$ value. All three AMPs caused some degree of nonmonotonicity in lipid chain order, indicating that lipid chain order, unlike $K_{\text{C}}$, may be unrelated to their different bacterial killing efficacies.

**Figure 7.**
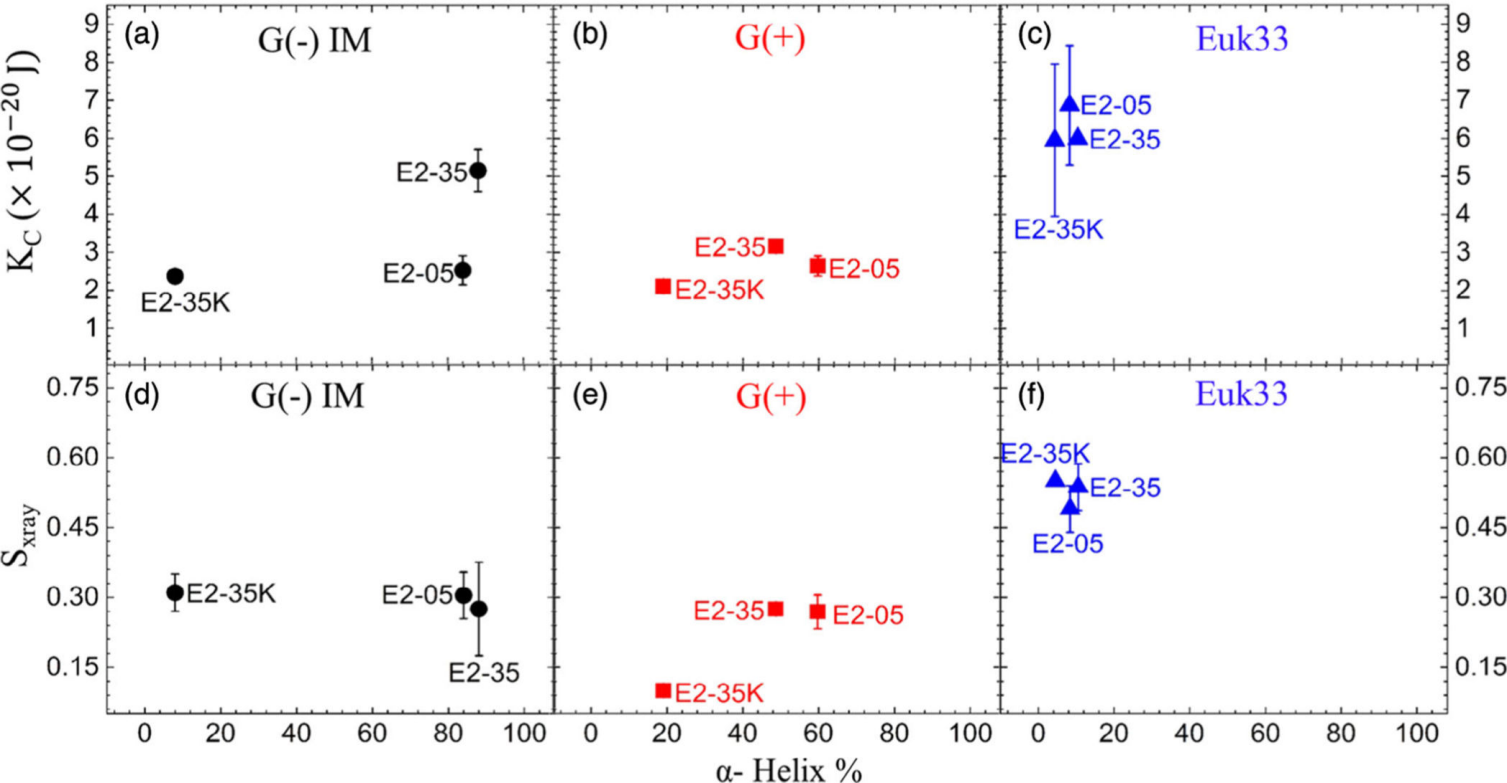
% α-helix versus bending modulus ($K_{\text{C}}$) of AMPs in a). G(−) IM LMMs (black circles), b) G(+) LMMs (red squares), and c) Euk33 LMMs (blue triangles). % α-helix versus chain order parameter ($S_{\text{X-ray}}$) of AMPs in d). G(−) IM LMMs (black circles), e). G(+) LMMs (red squares), and f). Euk33 LMMs (blue triangles). The lipid/peptide molar ratio is 75:1. The standard deviations are from duplicate or triplicate samples.

It is of interest to investigate possible correlations of peptide secondary structure with elastic parameters. Figure 7a shows that the $K_{\text{C}}$ values for E2–35 and E2–05 are quite different yet; their helicity values are similar. In addition, the helicity values for E2–35K and E2–05 are very different but their $K_{\text{C}}$ values are almost the same. In Figure 7b,c, there is no clear trend in $K_{\text{C}}$ versus helicity. Therefore, we suggest that bending rigidity (either softer or more rigid membranes) is not correlated with secondary structure.

Figure 7d–f shows a positive correlation of acyl chain order with increasing α-helicity only in G(+) LMMs. Changes in lipid acyl chain order often parallel the trend of changes in bending modulus, but this is not always the case as shown by Boscia et al. (Table 3 in ref. [70]). This suggests that changes in $K_{\text{C}}$ may be more relevant than changes in acyl chain order for these E2 peptides.

### 2.5. Membrane Structural Results

**Figure 8.**
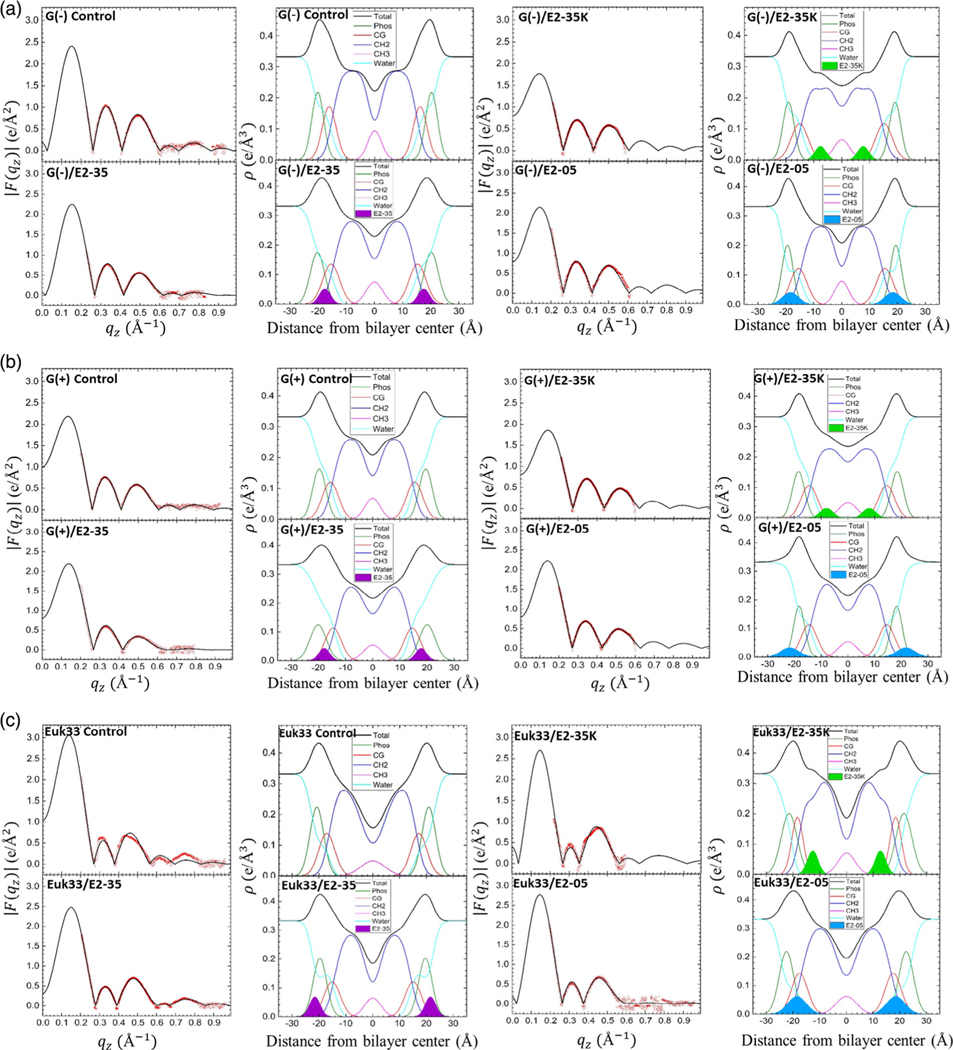
$\left|F\left(q_{z}\right)\right|$ (columns 1 and 3) and EDPs (columns 2 and 4) for **a.** G(−) IM LMMs, **b.** G(+) LMMs, and c. Euk33 LMMs in the presence of E2–35, E2–35K, or E2–05. The red points are experimental data and the black line is the SDP model fit to the data. Component groups in EDPs: phosphate + external headgroup (Phos, green), carbonyl–glycerol (CG, red), CH2 (dark blue), CH3 (magenta), water (cyan), total (black), E2–35 (filled purple), E2–35K (filled lime), and E2–05 (filled dark cyan). Lipid/peptide molar ratio is 75:1.

In this section, we explore correlations between the peptide location in the bilayers and structural changes of the LMMs with the bacterial killing efficiency of three peptides. Figure 8 displays form factors $\left|F\left(q_{z}\right)\right|$ and electron density profiles (EDPs) of the three LMMs containing 75:1 lipid/peptide molar ratio of E2–35, E2–05, or E2–35K, obtained by fitting XDS form factor data with the scattering density profile (SDP) program.^[71]^ This program considers volumes of the lipid, peptides, and component groups in the bilayer and the number of electrons for each component. We fit the form factors by placing a Gaussian envelope for the peptide in three different locations: headgroup, hydrocarbon, or half headgroup–half hydrocarbon. As shown in Figure 8, there was generally an excellent fit of the SDP bilayer model to the XDS form factor data, where the resulting EDPs are typical of fully hydrated membranes. The component groups in the EDPs are Phos (phosphate plus outer headgroup), CG (carbonyl/glycerol), CH2 (methylene hydrocarbon region which also contains CH groups), CH3 (terminal methyl group), water (which fills in the volumes around the other groups so that the total volume probabilities sum to one), and total (the sum of all the component groups). The combined Phos and CG peak–peak distance ($D_{\text{HH}}$) and the hydrocarbon full-width at half-maximal ($2D_{\text{C}}$) are two measures of the membrane thickness. The EDP also determines the area per lipid molecule ($A_{\text{L}}$) when the lipid and peptide volumes are measured in a separate experiment. We found that measuring the individual lipid volumes and combining them gave a more accurate molecular volume when compared to MD simulation^[72]^ than measuring a mixture of three of four lipids in the densimeter. A summary of the XDS structural results for the three LMMs used in this study interacting with E2–35, E2–35K, and E2–05 is shown in Table 1.

Our XDS data reveal that both E2–35 and E2–05 locate in the headgroup region of G(−) and G(+) LMMs, whereas E2–35K locates deeper into the hydrocarbon region of both bacterial LMMs as shown in Figure 8a,b. Note that E2–35K is not as effective in killing bacteria as the other two as shown in Figure 3, so headgroup location correlates with efficient bacterial killing. The cause of the headgroup location in E2–35 (8 Rs) and E2–05 (12 Rs) could be their high R content and interior location of E2–35K could be because of its K content. The free energy barrier experienced by a K crossing the membrane is strikingly similar to that of a R (to within ≈2 kcal mol^−1^), despite the two having different chemistries, H-bonding capability, and hydration free energies that differ by ≈10 kcal mol^−1^.^[73]^ While R and K are nearly equal in hydrophilicity,^[74]^ R contains two additional nitrogens, giving the guanidinium moiety the capacity for up to six hydrogen bonds.^[75]^ This unique ability of R leads to various R-phosphate complexes. A recent simulation study by Allolio et al. has revealed that charged nonaarginine (${\text{R}}_{9}$) side chains bind to lipid headgroups, primarily at the negatively charged phosphates.^[76]^ Further, Menger et al. have shown that poly-l-lysine, despite its polycationic character, can pass through a lipid bilayer when complexed with an anionic lipid present in the vesicle wall.^[77]^ Therefore, it is more likely for the Arg-rich peptides E2–35 and E2–05 to be in the headgroup region, where they can bind to the phosphate group and to negatively charged lipid headgroups (POPG and TOCL) of G(−) and G(+) LMMs. By contrast, K-rich peptide E235K locates in the hydrocarbon core of all three LMMs.

To verify this headgroup location, we also carried out NR on one sample, E2–35, in a G(−) LMM tethered bilayer. NR is more sensitive to the peptide location due to the high scattering length density contrast between ${\text{H}}_{2}\text{O}$ and ${\text{D}}_{2}\text{O}$. As shown in Figure S3, Supporting Information, E2–35 is located primarily in the headgroup region, confirming the location determined using XDS. As shown in Table 1, all three peptides reduce the membrane thickness ($2D_{\text{C}}$ and $D_{\text{HH}}$) in G(−) and G(+) membranes regardless of location in the bilayer. Similarly, all three peptides increase the $A_{\text{L}}$ in G(−) and G(+) LMMs. Therefore, this strongly suggests that membrane thickness and area/lipid changes are uncorrelated with bacterial killing efficiency.

In Euk33 LMMs, the peptides E2–35 and E2–05 locate in the headgroup region, while E2–35K locates in the hydrocarbon interior, similar to their locations in G(−) and G(+) LMMs. This suggests that an E2 peptide in the hydrocarbon region is less effective at killing both eukaryotic and bacterial cells. Table 1 shows either no change or no clear trend in changes in membrane thickness in Euk33 LMMs with all three peptides. E2–05 and E2–35, which are both toxic to eukaryotic cells, have opposite effects from each other on membrane thickness ($2D_{\text{C}}$), and also opposite effects on $A_{\text{L}}$.

## 3. Conclusions

In this detailed structure/function study, we have found that three primarily helical E2 peptides consisting of R, W, and V (E2–35, E2–05, and E2–71) are efficacious at killing both G(−) and G(+) bacteria in in vitro assays. On average, the 14-mer (E2–32), with smaller helical content, is less effective than AMPs containing 16–22 amino acids. Far less efficacious and also less helical is E2–35K, where all 8 Rs in E2–35 are replaced with Ks. Also, less efficacious and less helical is E2–75, where a single hydrophobic V residue together with 3 Ws near the interfacial region in the helical wheel diagram is sufficient to disrupt helicity. We also found that α-helicity is correlated with toxicity of both RBCs and WBCs. AMP α-helical content decreases as a function of lipid composition as G(−)>G(+)>Euk33 LMMs. AMP α-helical content is also correlated with increased membrane rigidity. Nonmonotonic changes in $K_{\text{C}}$ are correlated with efficient bacterial killing, but not toxicity. Lipid acyl chain order does not appear to be involved in the bacterial killing mechanism or eukaryotic toxicity. Our structural study revealed that an AMP headgroup location is required for efficient bactericidal activity and toxicity because both E2–35 and E2–05 are located in the lipid headgroup region, while E2–35K is located in the hydrocarbon interior.$A_{\text{L}}$ and membrane thickness changes are not correlated with bacterial killing or eukaryotic toxicity. We suggest that the 16-mer E2–35 is a better candidate for an AMP than the 22-mer E2–05 because their bacterial killing efficacies and biophysical properties are similar, but E2–35 is shorter, so it is less expensive to produce.

## 4. Experimental Section

### Materials:

The synthetic lyophilized lipids 1-palmitoyl-2-oleoyl-*sn*-glycero-3-phosphoethanolamine (POPE), 1-palmitoyl-2-oleoyl-*sn*-glycero-3-phospho-(10-rac-glycerol) sodium salt (POPG), 10,30-bis [1,2-dioleoyl-*sn*-glycero-3-phospho]-*sn*-glycerol sodium salt (TOCL, i.e., cardiolipin), 1-palmitoyl-2-oleoyl-*sn*-glycero-3-phosphocholine (POPC), egg sphingomyelin (ESM), and 1,2-dioleoyl-3-trimeathylammoniumpropane chloride salt (DOTAP) were purchased from Avanti Polar Lipids (Alabaster, AL) and used as received. Cholesterol was from Nu-Chek Prep (Waterville, MN). HPLC-grade organic solvents were purchased from Sigma–Aldrich (St. Louis, MO). Lipid stock solutions in chloroform were combined to create lipid mixtures in molar ratios mimicking the G(−) IM: POPE/POPG/TOCL (7:2:1 molar ratio), G(+) membrane: POPG/DOTAP/POPE/TOCL (6:1.5:1.5:1),^[78]^ and eukaryotic membrane, Euk33: POPC/ESM/POPE/cholesterol (15:4:1:10) (33 mol% cholesterol).^[79]^ Bacterial cation-adjusted Mueller Hinton Broth (MHB2), Test Condition Media, RPMI, fetal bovine serum (FBS), and phosphate-buffered saline (PBS) were obtained from Millipore Sigma (St. Louis, MO). Formaldehyde was obtained from Thermo Fisher (Waltham, MA). All peptides were purchased in lyophilized form (10 mg in a 1.5 mL vial) from Genscript (Piscataway, NJ) with HPLC/MS spectra corresponding to each designed primary sequence. The traditional antibiotics and colistin were purchased from Millipore Sigma (St. Louis, MO). Amino acid sequences of the peptides and their physical attributes are provided in Table 2.

### Antibacterial Assay:

Bacterial clinical isolates used for initial screening were anonymously provided by the clinical microbiology laboratory of the University of Pittsburgh Medical Center. Bacteria were stored at −80 °C and typically retrieved by obtaining single colonies on agar plates prior to subsequent liquid broth culture. Suspensions of test bacteria were prepared from the log phase of growth by diluting overnight cultures at 1:100 with fresh cation-adjusted MHB and incubating for an additional 3–4 h. Bacteria were spun at 3000 g for 10 min. The pellet was resuspended in Test Condition Media to determine bacterial turbidity using a Den-1B densitometer (Grant Instruments, Beaver Falls, PA) at 0.5 McFarland units corresponding to 108 CFU mL^−1^.

To examine antibacterial activity, we used minor modifications of a standard growth inhibition assay endorsed by the Clinical and Laboratory Standards Institute (CLSI), as previously described.^[80]^ Bacteria were incubated with each of the indicated peptides in MHB2. The bacterial cells were kept in an incubator for 18 h at 37 °C, which is linked to a robotic system that feeds a plate reader every hour with one of 8 × 96-well plates. This setup allows the collection of growth kinetic data at A 570 (absorbance at 570 nm) to examine growth inhibition in real-time (BioTek Instruments, Winooski, VT). We defined MIC as the minimum peptide concentration that completely prevented bacterial growth, demonstrated by a flat (horizontal line) growth curve at A570. The assays are typically repeated a second time. If the MIC differs from the first assay, a third experimental trial is performed to confirm the MIC.

### Determination of Toxicity to Mammalian Cells:

Toxicity to primary cells was examined using human RBCs and peripheral mononuclear cells (PBMCs or WBCs) as previously described.^[45,81]^ Briefly, RBCs and WBCs were separated by histopaque differential centrifugation using blood anonymously obtained from the Central Blood Bank (Pittsburgh, PA). For the RBC lysis assay, the isolated RBCs were resuspended in PBS at a concentration of 5%. The peptides were serially diluted twofold in 100 μL of PBS before adding 100 μL of 5% RBC to a final dilution of 2.5% RBC to ensure that the A570 of hemoglobin does not saturate the plate reader. In parallel, the RBCs were osmotically burst with water at different concentrations to generate a standard curve of RBC lysis. Three technicians independently conducted experiments to ensure reproducibility.

Human WBCs were treated with each selected peptide in RPMI and 10% FBS and incubated for 1 h at 37 °C. The cells were then immediately washed with PBS at 1000 g for 7 min while in a round-bottom 96-well plate. After resuspension in PBS, we added fixable blue live/dead stain from Life Technologies according to the manufacturer’s instructions. The cells were again washed and resuspended in PBS to remove nonspecific stain and then fixed with 4% formaldehyde for 1 h. After washing with PBS, the samples were stored at 4 °C overnight (in the dark) before examination by flow cytometry using the Novocyte flow cytometer (Agilent Technologies, Santa Clara, CA). Peptide-treated cells were compared with untreated cells for dye incorporation, and data were analyzed using the Novocyte analytical software. Dye incorporation was quantified as percent toxicity directly determined by distinguishing live from dead populations,^[81]^ which was plotted using GraphPad (Prizm software, San Diego, CA).

### CD:

Unilamellar vesicles (ULVs) of ≈600 Å diameter were prepared using an extruder (Avanti Polar Lipids, Alabaster, AL). A 100 μL of 20 mg mL^−1^ lipid in 15 mM PBS was extruded 21 times through a single Nucleopore filter of size 500 Å using 0.2 mL Hamilton syringes. The final lipid concentration of lipid in the ULVs was 15 mg mL^−1^ as determined gravimetrically. Concentrated ULVs were added to 3 mL of 10 μM peptide in 15 mM PBS at pH 7 to create lipid/peptide molar ratios between 0:1 and 70:1. The samples remained at room temperature for ≈16 h before the CD measurement. Data were collected in 3 mL quartz cuvettes using a Jasco 1500 CD spectrometer at 37 °C in the Center for Nucleic Acids Science and Technology (CNAST) at Carnegie Mellon University. The samples were scanned from 200 to 240 nm 20 times and the results were averaged. The temperature was controlled at 37 °C via a Peltier element and water circulation through the sample compartment. Nitrogen gas was used at a flow rate between 20 and 25 ft^3^ h^−1^. OriginPro 2019 (OriginLab, Northampton, MA) was used to carry out a Levenberg–Marquardt least squares fit of the smoothed ellipticity traces to four secondary structural motifs representing α-helix, β-sheet, β-turn, and random coil.^[20,82,83]^ This analysis gives a percentage match of each secondary structural motifs to the total sample ellipticity. Instrument ellipticity (ε) was converted to mean residue ellipticity using MRE $\left({\text{deg cm}}^{2}\;{\text{dmol}}^{-1}\right)=\varepsilon 10^{4}/N$, where N=# amino acids and peptide concentration was always 10 μM.

### Low-Angle XDS:

Oriented samples consisting of stacks of approximately ≈1800 bilayers were prepared using the well-established “rock and roll” method.^[84]^ 4 mg of lipids and peptides in organic solvent, chloroform: methanol (2:1, v/v) or trifluoroethanol:chloroform (1:1, v/v), were deposited onto a Si wafer (15 mm × 30 mm) inside a fume hood. After rapid evaporation while rocking the substrate, an immobile film formed which was then further dried inside the fume hood for two hours, followed by overnight drying under vacuum to evaporate residual organic solvent. The samples were trimmed to occupy 5 mm × 30 mm along the center of the Si substrate. The sample was mounted into a thick-walled X-ray hydration chamber which provides greater than 99% relative humidity. Even greater RH was obtained with a Peltier element underneath the Si wafer which, by cooling the sample relative to the vapor, allowed tuning the D spacing up to full hydration.^[85]^ Low-angle XDS (LAXS) data from oriented, fully hydrated samples were obtained at the ID7A line at the Center for High Energy X-ray Sciences (CHESX, Ithaca, NY) on three separate trips using X-ray wavelengths of 0.8785, 0.8434, and 0.8819 Å and sample-to-detector (*S*)-distances of 546.5, 410, and 390.5 mm, with an Eiger 4M detector. Measurements were carried out in the fluid phase at 37 °C. The flat silicon wafer was rotated from −1 to 6 ° during the data collection at CHESS. The background was collected by setting the X-ray angle of incidence to −1.7°, where sample scattering does not contribute to the image. For data analysis, backgrounds were first subtracted to remove extraneous air and mylar scattering and the images were laterally symmetrized to increase the signal-to-noise ratio. As the sample nears full hydration, membrane fluctuations occur which produce “lobes” of diffuse X-ray scattering data.^[86]^ The fluctuations are quantitated by measuring the fall-off in lobe intensity in the lateral q_r_ direction. The fitting procedure is a nonlinear least squares fit that uses the free energy functional from liquid crystal theory^[87]^

(1) $$f=\frac{\pi }{NL_{r}^{2}}\int r\;\text{d}r\sum_{n=0}^{N-1}\left\{K_{\text{C}}{\left[{\text{Δ}}_{\text{r}}^{2}u_{n}(r)\right]}^{2}+B{\left[u_{n+1}(r)-u_{n}(r)\right]}^{2}\right\}$$

where N is the number of bilayers in the vertical (Z) direction, $L_{\text{r}}$ is the domain size in the horizontal (r) direction, and $K_{\text{C}}$ is the bending modulus. In our experiment, $K_{\text{C}}$ describes the bending of a single bilayer, $u_{n}$ is the vertical membrane displacement, and B is the compressibility modulus. We can obtain $K_{\text{C}}$ and B independently. A higher $K_{\text{C}}$ indicates a stiffer membrane, and a lower $K_{\text{C}}$ corresponds to a softer membrane.

### Wide-Angle XDS:

Wide-angle XDS (WAXS) was obtained at CHESX. In order to obtain WAXS data, the same sample that was hydrated for LAXS is X-rayed with a glancing angle of incidence, instead of a rotation of the sample. Two exposures are taken at angles of X-ray incidence $\alpha =+0.3^{\circ }$ and $\alpha =-0.3^{\circ }$, where the negative angle image is then subtracted from the positive angle image. Both are 30 s scans. The subtraction procedure removes extraneous scatter due to the mylar chamber windows and shadows. The chain–chain correlation appears as strong diffuse scatter projecting upward circularly from the equator; the fall-off in intensity yields information about chain order. To obtain an $S_{\text{X-ray}}$ order parameter, the intensity is first integrated along its trajectory, and then fit to a wide-angle liquid crystal theory.^[88]^ The chain scattering model assumes long thin rods that are locally well aligned along the local director $\left(n_{\text{L}}\right)$, with orientation described by the angle β. While acyl chains from lipids in the fluid phase are not long cylinders, the model allows the cylinders to tilt (β) to approximate chain disorder. From the fit of the intensity data using a MATLAB computer program, we obtain $S_{\text{X-ray}}$ using Equation (2)

(2) $$S_{\text{X-ray}}=\frac{1}{2}\left(3\langle \cos^{2}\text{β}\rangle -1\right)$$

We also obtain the RMSE (root mean square error), which reports the goodness of the fit.

### NR:

Neutrons have greater contrast in scattering length density for peptides compared to lipids than X-ray, making NR a powerful technique to determine the exact position of the peptide inside the membrane. 6 mg G(−) IM lipid/peptide mixtures were cosolubilized in organic solvent, dried under vacuum, and hydrated for 1–2 h via bath sonication in 1.2 mL 2 M NaCl. A single membrane bilayer was deposited onto a lipid-tethered gold-covered $3^{\prime \prime }$ silicon wafer over ≈2 h using the vesicle fusion method.^[89]^ NR data were collected at the NGD-MAGIK reflectometer at the NIST Center for Neutron Research (Gaithersburg, MD) over a momentum transfer range 0–0.25 Å^−1^. Six hour scans were collected in either ${\text{H}}_{2}\text{O}$ or ${\text{D}}_{2}\text{O}$ at 37 °C. Data were analyzed at NIST; 1D-structural profiles showing the component volume occupancy are parameterized using a continuous distribution model using Refl1D software packages.^[90]^

## Supplementary Material

SM

## Figures and Tables

**Table 1.** Summary of structural results from XDS and the net charge/residue.

Sample (lipid/peptide (75:1))	Area/lipid $A_{\text{L}}\;\left[{\text{Å}}^{2}\right]$ (±1.0)	$D_{\text{HH}}\;[\text{Å}]$ (±0.5)	$2D_{\text{c}}\;[\text{Å}]$ (±0.5)	Net charge/residue
G(−)IM/control	70.8	39.2	29.1	–
G(−)IM/E2-35	75.5	38.4	27.3	−0.178
G(−)IM/E2-35K	76.1	37.9	28.1	−0.178
G(−)IM/E2-05	72.5	38.2	28.5	−0.127
G(+)/control	73.4	38.5	28.9	–
G(+)/E2-35	79.0	37.5	26.9	−0.209
G(+)/E2-35K	82.9	36.6	26.6	−0.209
G(+)/E2-05	78.9	36.5	26.9	−0.150
Euk33/control	64.0	40.3	32.0	–
Euk33/E2-35	73.6	39.0	28.0	0.006
Euk33/E2-35K	63.5	40.0	34.4	0.006
Euk33/E2-05	62.2	39.7	33.1	0.007

**Table 2.** Amino acid sequences of the peptides and their physical attributes. The charged residues are in red type.

Peptide	Amino acid sequence	#AA	Charge	μH	H	μH/H
E2-32	RR VW R WV RR WW RR V	14	+7	0.849	0.399	2.13
E2-35	RR VW R WV RR VW R WV RR	16	+8	0.736	0.363	2.03
E2-35K	KK VW K WV KK VW K WV KK	16	+8	0.729	0.372	1.96
E2-05	RR VW RR V RR VV RR W RR WV RR VV	22	+12	0.779	0.144	5.41
E2-71	RR VW RR V RR VW RR V RR VV RR VW	22	+12	0.777	0.144	5.39
E2-75	RR VW RR V RR VW RR V RR VW RR VV	22	+12	0.798	0.144	5.54

## Data Availability

The data that support the findings of this study are available from the corresponding author upon reasonable request.
