# Supplementary material for: Novel Helical Trp- and Arg-Rich Antimicrobial Peptides Locate Near Membrane Surfaces and Rigidify Lipid Model Membranes
Source: Adv Nanobiomed Res. Author manuscript; Available in PMC 2023 Jul 20. (PMC10358585; doi:10.1002/anbr.202300013)
Supplement: SM [file NIHMS1912392-supplement-SM.pdf]

Supplementary Information

Novel helical Trp- and Arg-rich antimicrobial peptides locate near membrane surfaces and rigidify lipid model membranes

Saheli Mitra, Mark Coopershlyak, Yunshu Li, Bhairavi Chandersekhar, Rachel Koenig, Mei-Tung Chen, Brandt Evans, Frank Heinrich, Berthony Deslouches, Stephanie Tristram-Nagle

For comparison studies of hydrophobic moment (μH), hydrophobicity (H) and μH /H we used the HeliQuest website (https://heliquest.ipmc.cnrs.fr) to calculate these quantities. HeliQuest relies on two primary references for these calculations.[1, 2] The quantities H and μH are unitless in these two references.

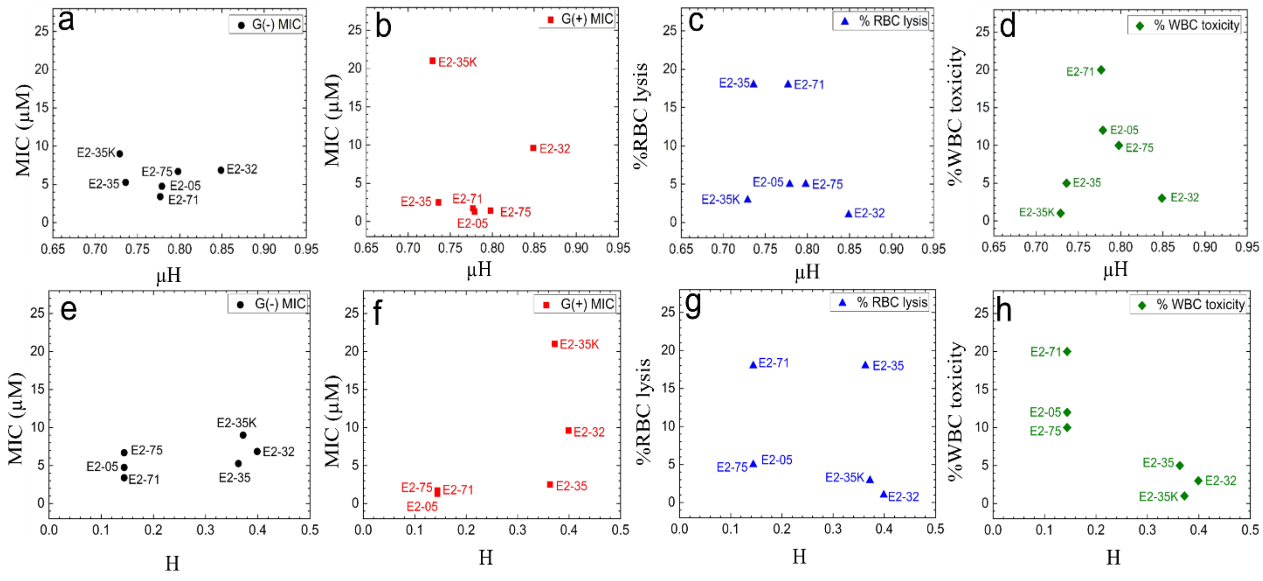

Figure S1. **a.** MIC vs. hydrophobic moment (μH) of E2-peptides in G(-) LMMs. **b.** MIC vs. μH in G(+) LMMs. **c.** % RBC lysis vs. μH of E2 peptides and **d.** %WBC toxicity. **e.** MIC vs. hydrophobicity (H) of E2-peptides in G(-) LMMs. **f.** MIC vs. H in G(+) LMMs. **g.** %RBC lysis vs. H of E2 peptides at 32 μM and **h**. %WBC toxicity at 16 μM. The six E2-peptides are E2-32, E2-35, E2-35K, E2-05, E2-71 and E2-72.

Plots of μH vs. MIC did not show a clear correlation of μH with bacterial toxicity for either G(-) or G(+) (Figure S1a,b). Plots of H vs. MIC could be interpreted as a positive correlation between H and MIC, i.e., poorer efficacy with increasing hydrophobicity (Figure S1e,f). Plots of μH vs. toxicity showed a maximum toxicity at ~0.72 to 0.78 μH for both % RBC lysis and WBC toxicity

(Figure S1c,d). Plots of H vs. toxicity, showed either no correlation in % RBC lysis (Figure S1g) or a negative correlation in % WBC toxicity (Figure S1h), indicating less toxicity with higher hydrophobicity. Our H results were opposite to one systematic investigation which reported that hemolytic activity (% RBC lysis) was positively correlated with increasing hydrophobicity, while MIC displayed a "sweet spot" of hydrophobicity vs. efficacy.[3] In that study, peptide aggregation decreased antibacterial activity as hydrophobicity increased beyond an ideal value.[3] In another study, antibacterial activity increased above a threshold value of hydrophobicity.[4] In a third study, as the hydrophobicity increased, antibacterial activity increased.[5]

Perhaps less controversial than hydrophobicity is the µH/H ratio of an AMP vs. bacterial and eukaryotic toxicity, where hydrophobicity acts to modulate amphipathicity. The use of µH/H is a way to normalize the hydrophobic moment with hydrophobicity. In Figure S2a,b, as µH/H increases, MIC decreases in both G(-) and G(+) bacteria. In addition, toxicity in eukaryotic cells also increases (Figure S2 c,d). Therefore, we suggest that there could be a "sweet spot" with a value of µH/H (4-5) where good bactericidal activity and tolerable toxicity are achieved. Compared to the literature, most studies do not calculate the µH/H ratio. Two studies reported µH and H values separately and our lab calculated their µH/H ratio and compared it to their reported MIC values.[6, 7] Ignoring very large MIC values, no clear trend in µH/H ratio vs. MIC was observed in either work. The reported inconsistencies in the literature when comparing µH, H or µH/H to MIC or toxicity could be due to different ways of calculating µH and H, using different hydrophobicity scales or assuming that the peptide is 100 % helical. While these calculations are an interesting exercise, we suggest that changes in these calculated properties are not as important as secondary structural changes of the AMPs, and elastic changes in the LMMs, shown in the main paper.

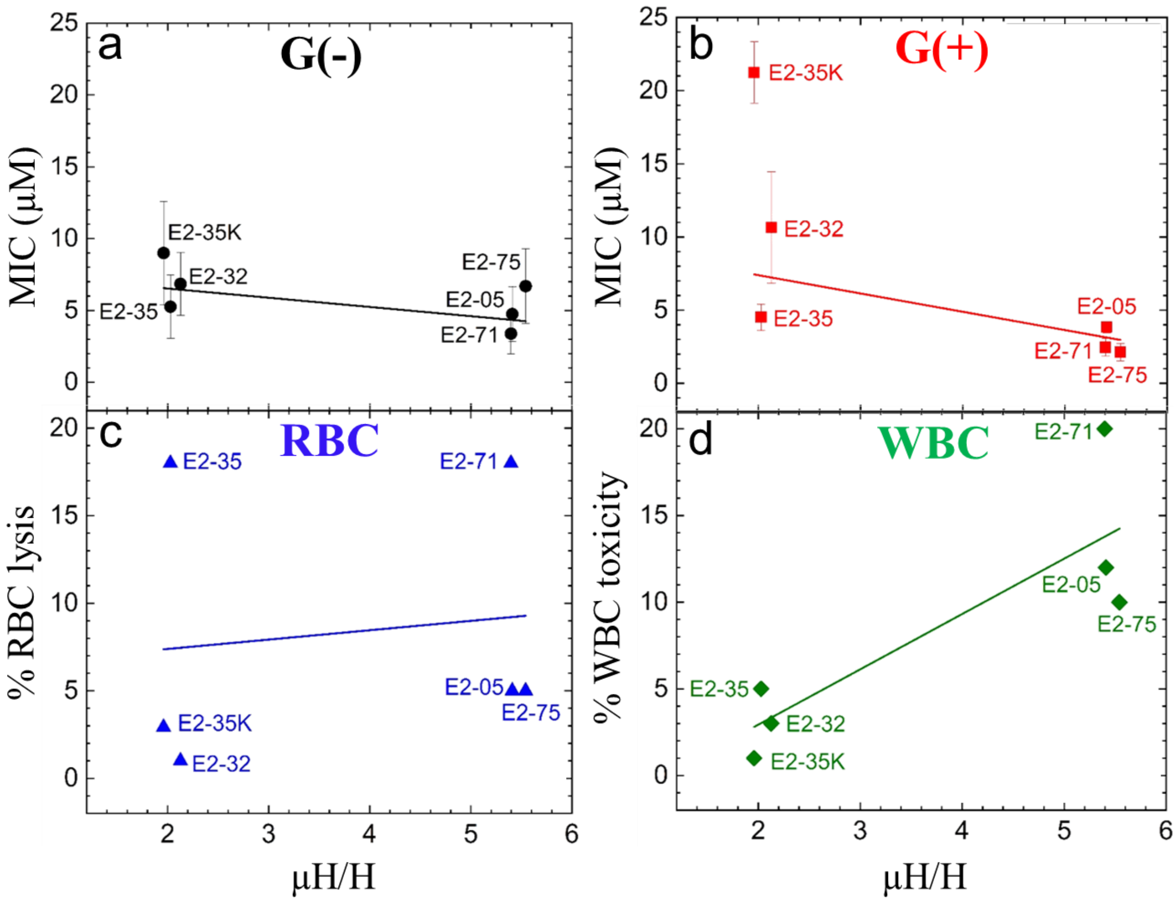

Figure S2. a. MIC vs. μH/H for AMPs in G(-) bacteria (black circles). **b.** MIC vs. μH/H for AMPs in G(+) bacteria (red squares). **c.** % RBC lysis vs. μH/H of the AMPs (blue triangles). **d.** % WBC toxicity vs. μH/H of the AMPs (green diamonds). The standard deviations were calculated as in Figure 2. Straight lines are linear fits to the data.

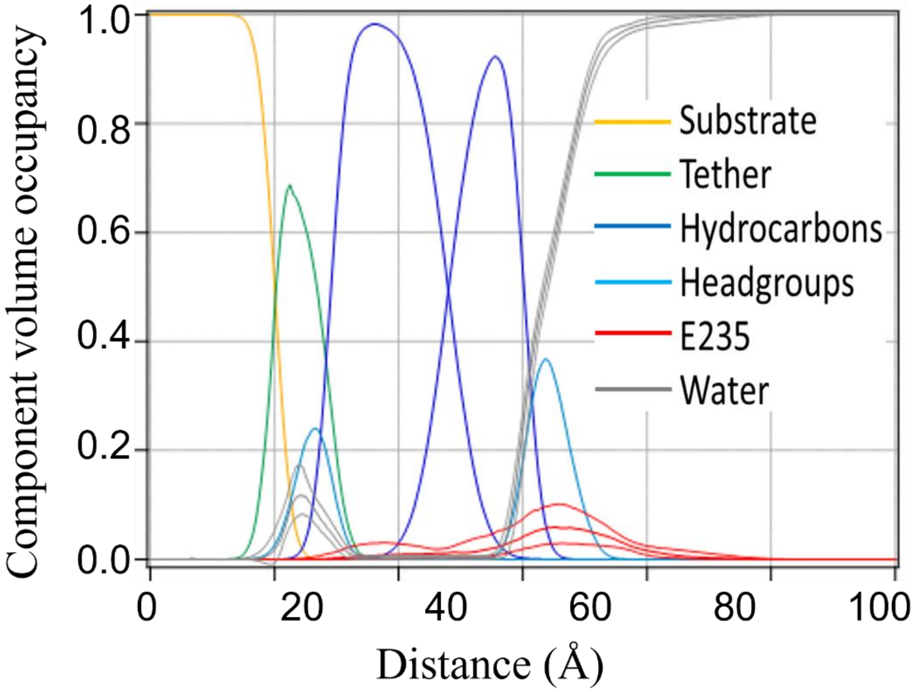

Figure S3. Neutron reflectivity (NR) component volume occupancy of E2-35 in a single tethered bilayer of G(-) IM. Component volumes: gold-covered silicon substrate (yellow), tether (green), headgroups (cyan), hydrocarbons (blue), E2-35 (red), water (grey). The pink lines represent the 68 % confidence limit of the composition-space fit.

Tables S1 – S18 summarize secondary structural results (% α-helix, β-sheet, β-turn and random coil) for six E2 peptides in three LMMs. R^2 indicates the goodness of fit. Std. devs. were generally ~5-7 % of the values shown.

Table S1: E2-32 CD results of secondary structure in G(-) IM

G(-) IM/Peptide molar ratio	α-helix (%)	β-sheet (%)	β-turn (%)	Random (%)	R^2
0:1	0	45	0	55	0.97
5:1	16	43	0	42	0.92
10:1	11	59	0	30	0.84
20:1	44	9	0	47	0.88
30:1	73	15	12	0	0.55
50:1	68	15	0	17	0.82
70:1	65	18	0	17	0.85

Table S2: E2-32 CD results of secondary structure in G(+) LMMs

G(+) /Peptide molar ratio	α-helix (%)	β-sheet (%)	β-turn (%)	Random (%)	R^2
0:1	0	45	0	55	0.97
10:1	24	39	0	37	0.94
20:1	25	38	0	37	0.89
30:1	21	41	0	38	0.95
50:1	23	34	0	43	0.95
70:1	23	27	6	44	0.94

Table S3: E2-32 CD results of secondary structure in Euk33 LMMs

Euk33 /Peptide molar ratio	α-helix (%)	β-sheet (%)	β-turn (%)	Random (%)	R^2
0:1	0	45	0	55	0.97
5:1	0	43	0	57	0.97
10:1	0	44	0	56	0.98
30:1	4	40	0	56	0.96
70:1	18	29	0	53	0.83

Table S4: E2-35 CD results of secondary structure in G(-) IM LMMs

G(-) IM/Peptide molar ratio	α-helix (%)	β-sheet (%)	β-turn (%)	Random (%)	R^2
0:1	0	54	0	46	0.97
5:1	0	53	47	0	0.76
10:1	88	9	3	0	0.87
15:1	78	3	19	0	0.76
20:1	56	10	0	34	0.84
25:1	50	9	10	31	0.80
30:1	43	11	13	33	0.81
50:1	38	25	1	36	0.83
70:1	39	17	8	36	0.78

Table S5: E2-35 CD results of secondary structure in G(+) LMMs

G(+) IM/Peptide molar ratio	α-helix (%)	β-sheet (%)	β-turn (%)	Random (%)	R^2
0:1	0	54	0	46	0.97
5:1	4	84	2	10	0.98
10:1	45	16	39	0	0.78
20:1	49	14	19	18	0.80
30:1	49	7	21	23	0.64
50:1	46	16	10	28	0.77
70:1	43	14	13	30	0.75

Table S6: E2-35 CD results of secondary structure in Euk33 LMMs

Euk33/Peptide molar ratio	α-helix (%)	β-sheet (%)	β-turn (%)	Random (%)	R^2
0:1	0	54	0	46	0.97
5:1	0	49	0	51	0.97
10:1	5	44	0	51	0.95
20:1	10	37	0	53	0.93
30:1	0	53	0	47	0.98
70:1	6	42	0	52	0.81

Table S7: E2-35K CD results of secondary structure in G(-) IM LMMs

G(-) IM/Peptide molar ratio	α-helix (%)	β-sheet (%)	β-turn (%)	Random (%)	R^2
0:1	0	47	0	53	0.93
10:1	0	39	7	54	0.95
20:1	5	54	0	41	0.92
30:1	8	45	3	44	0.96
50:1	2	63	0	35	0.77
70:1	4	54	0	42	0.96

Table S8: E2-35K CD results of secondary structure in G(+) LMMs

G(+)/Peptide molar ratio	α-helix (%)	β-sheet (%)	β-turn (%)	Random (%)	R^2
0:1	0	47	0	53	0.93
10:1	5	71	0	24	0.84
20:1	9	49	1	41	0.94
30:1	19	46	0	35	0.92
50:1	16	43	0	41	0.91
70:1	17	43	0	40	0.93

Table S9: E2-35K CD results of secondary structure in Euk33 LMMs

Euk33/Peptide molar ratio	α-helix (%)	β-sheet (%)	β-turn (%)	Random (%)	R^2
0:1	0	47	0	53	0.93
10:1	0	46	0	54	0.98
20:1	0	45	0	55	0.98
30:1	2	44	0	54	0.96
70:1	4	49	0	47	0.77

Table S10: E2-05 CD results of secondary structure in G(-) IM LMMs

G(-) IM/Peptide molar ratio	α-helix (%)	β-sheet (%)	β-turn (%)	Random (%)	R^2
0:1	3	37	1	59	0.99
5:1	13	18	18	51	0.98
10:1	29	33	0	38	0.96
20:1	51	46	3	0	0.97
50:1	84	0	8	8	0.83
70:1	73	4	17	6	0.90

Table S11: E2-05 CD results of secondary structure in G(+) LMMs

G(+)/Peptide molar ratio	α-helix (%)	β-sheet (%)	β-turn (%)	Random (%)	R^2
0:1	3	37	1	59	0.99
5:1	21	41	0	38	0.94
10:1	52	42	2	4	0.91
20:1	36	43	0	21	0.93
30:1	34	36	2	28	0.85
50:1	37	37	1	25	0.88
70:1	33	34	8	25	0.88

Table S12: E2-05 CD results of secondary structure in Euk33 LMMs

Euk33/Peptide molar ratio	α-helix (%)	β-sheet (%)	β-turn (%)	Random (%)	R^2
0:1	3	37	1	59	0.99
5:1	3	37	0	60	0.99
10:1	4	36	0	60	0.99
30:1	8	35	0	57	0.97
70:1	8	40	1	51	0.75

Table S13: E2-71 CD results of secondary structure in G(-) IM LMMs

G(-) IM/Peptide Molar Ratio	α-helix (%)	β-sheet (%)	β-turn (%)	Random (%)	R^2
0:1	10	33	1	55	0.97
5:1	17	35	0	48	0.97
10:1	18	49	0	33	0.94
20:1	47	49	5	0	0.84
30:1	74	4	21	0	0.47
50:1	80	19	1	0	0.85
70:1	70	25	5	0	0.83

Table S14: E2-71 CD results of secondary structure in G(+) LMMs

G(+) /Peptide molar ratio	α-helix (%)	β-sheet (%)	β-turn (%)	Random (%)	R^2
0:1	10	33	1	55	0.97
5:1	16	42	0	41	0.96
10:1	44	49	5	3	0.94
20:1	24	46	0	30	0.96
30:1	36	31	1	32	0.93
50:1	36	34	0	29	0.92
70:1	50	27	0	23	0.91

Table S15: E2-71 CD results of secondary structure in Euk33 LMMs

Euk33 /Peptide molar ratio	α-helix (%)	β-sheet (%)	β-turn (%)	Random (%)	R^2
0:1	10	33	1	55	0.97
5:1	5	33	0	61	0.99
10:1	7	33	0	61	0.99
30:1	14	27	0	59	0.98
70:1	25	19	0	55	0.90

Table S16: E2-75 CD results of secondary structure in G(-) IM LMMs

G(-) IM/Peptide molar ratio	α-helix (%)	β-sheet (%)	β-turn (%)	Random (%)	R^2
0:1	1	40	0	59	0.99
5:1	7	43	0	50	0.93
10:1	6	48	0	46	0.90
20:1	8	50	0	42	0.82
30:1	6	63	0	31	0.85
50:1	4	55	0	41	0.91
70:1	0	66	0	34	0.94

Table S17: E2-75 CD results of secondary structure in G(+) LMMs

G(+) /Peptide molar ratio	α-helix (%)	β-sheet (%)	β-turn (%)	Random (%)	R^2
0:1	1	40	0	59	0.99
5:1	6	52	0	42	0.87
10:1	7	58	0	35	0.84
20:1	4	58	0	38	0.85
30:1	5	57	0	38	0.87
50:1	4	53	0	43	0.91
70:1	4	53	1	42	0.82

Table S18: E2-75 CD results of secondary structure in Euk33 LMMs

Euk33/Peptide molar ratio	α-helix (%)	β-sheet (%)	β-turn (%)	Random (%)	R^2
0:1	1	40	0	59	0.99
5:1	0	40	0	60	0.99
10:1	2	38	0	60	0.99
30:1	7	36	0	57	0.97
70:1	4	49	0	47	0.79

References for Supplementary Information

1. J. L. Fauchere, V. Pliska, *Eur J Med Chem* **1983,** *18*, 369.
2. D. Eisenberg, R. M. Weiss, T. C. Terwilliger, *Nature* **1982,** *299*, 371.
3. Y. X. Chen, M. T. Guarnieri, A. I. Vasil, M. L. Vasil, C. T. Mant, R. S. Hodges, *Antimicrobial Agents and Chemotherapy* **2007,** *51*, 1398.
4. M. Stark, L. P. Liu, C. M. Deber, *Antimicrobial Agents and Chemotherapy* **2002,** *46*, 3585.
5. Y. Rosenfeld, N. Lev, Y. Shai, *Biochemistry* **2010,** *49*, 853.
6. J. Zhang, R. Z. Sun, Z. W. Chen, C. Y. Zhou, C. B. Ma, M. Zhou, X. L. Chen, T. B. Chen, C. Shaw, L. Wang, *Biology-Basel* **2022,** *11*, 1263(1.
7. M. Dathe, T. Wieprecht, H. Nikolenko, L. Handel, W. L. Maloy, D. L. MacDonald, M. Beyermann, M. Bienert, *Febs Lett* **1997,** *403*, 208.
